# Association between root canal filling quality and apical periodontitis lesion volume: a cross-sectional cone-beam computed tomography study in a Chilean population

**DOI:** 10.1007/s00784-026-07085-2

**Published:** 2026-08-20

**Authors:** Rita A. Toloza-Espinoza, Pilar A. Araya-Cumsille, Marcela Torrealba, Valentina Valdivia, Milenka Filipic, Lúcio S. Gonçalves, José F. Siqueira, Flávio R. F. Alves

**Affiliations:** 1 Postgraduate Program in Dentistry, University of Grande Rio (UNIGRANRIO), Rua Professor José de Souza Herdy, 1160, Duque de Caxias, Rio de Janeiro, RJ 25071-202 Brazil; 2 Department of Endodontics, Universidad Andrés Bello, Santiago, Chile; 3 Department of Oral Radiology, Universidad Andrés Bello, Santiago, Chile; 4 Department of Dental Research, Faculty of Dentistry, Iguaçu University (UNIG), Nova Iguaçu, RJ Brazil

**Keywords:** Computed tomography, Periapical periodontitis, Root canal therapy, Root canal obturation, Tooth, nonvital

## Abstract

**Objectives:**

To investigate the association between root canal filling quality and the prevalence and volume of apical periodontitis (AP) lesions in a Chilean population using cone-beam computed tomography (CBCT).

**Materials and methods:**

This retrospective cross-sectional study evaluated CBCT scans acquired between 2021 and 2024 at a private radiology clinic in Santiago, Chile, for reasons unrelated to this investigation. A total of 830 root canal–treated teeth from 520 patients were analyzed. AP prevalence was recorded and lesion volume was quantified with the CBCT Periapical Volume Index. Fillings were classified as adequate or inadequate, and the canal-to-root diameter ratio was measured 1 mm short of the filling terminus. Associations, correlations, and odds ratios (OR) were analyzed at a 5% significance level.

**Results:**

Of the 830 teeth analyzed, AP was present in 37.3% (*n* = 310). Adequate fillings were associated with a lower lesion prevalence (OR = 0.514; CI 0.383-0.689; *p*< 0.001). Mean lesion volume was greater in inadequately (65.5 ± 52.3 mm³) than in adequately (50.3 ± 39.0 mm³; *p* < 0.05) filled teeth. Molars showed the highest prevalence (43.7%). A weak inverse correlation was found between the canal-to-root ratio and lesion volume (ρ = − 0.110; *p* < 0.05).

**Conclusions:**

AP affected over one-third of root canal–treated teeth in the study population. Adequate fillings and larger canal-to-root ratios were associated with a lower lesion prevalence and smaller lesion volumes.

**Clinical relevance:**

AP affected over one-third of root canal-treated teeth in this Chilean population. Adequate root canal fillings were significantly associated with a lower probability of AP and with smaller lesion volume. Larger canal-to-root ratios were associated with smaller lesion volumes.

## Introduction

Posttreatment apical periodontitis is an inflammatory condition resultant of persistent or secondary intraradicular infection, and characterizes an unsatisfactory outcome of the root canal treatment [1]. Epidemiological studies using two-dimensional radiographs across different populations have shown that a substantial proportion of root canal–treated teeth exhibit radiographic signs of apical periodontitis lesions (posttreatment disease), with prevalence ranging from 18% to over 60% depending on the study design, population, and diagnostic criteria [2–6]. In South America, two studies reported prevalence rates of posttreatment apical periodontitis of approximately 50% [7, 8].

The persistence of apical periodontitis has been strongly associated with the inadequate technical standard of root canal treatment, which has been mostly evaluated using the quality of filling as a surrogate [9]. Factors such as underfilling or overextension compromise the seal and allow residual or secondary intraradicular infection, which contribute to persistent periradicular inflammation [1, 10]. While the filling quality is a primary factor evaluated, other anatomical variables, such as canal-to-root diameter ratio (filled canal width relative to root width), may also influence periradicular health [11]. Exploring this relationship with modern three-dimensional imaging techniques can provide a more comprehensive understanding of the factors affecting the treatment outcome.

Traditionally, periradicular health has been assessed using two-dimensional radiographs. However, periapical radiographs have inherent limitations, including superimposition of anatomical structures, inability to detect early bone lesions, and restricted sensitivity in determining lesion size and extension [12]. Cone-beam computed tomography (CBCT) overcomes these shortcomings by enabling three-dimensional visualization and accurate volumetric quantification of apical periodontitis lesions [13–15].

Most CBCT-based epidemiological studies on periradicular health have been conducted in European populations [16–20]. To date, no study has evaluated the periradicular status and apical periodontitis lesion volume in root canal–treated teeth using CBCT in Chile. Moreover, previous CBCT studies have not explored the correlation between the periradicular status and the canal-to-root diameter ratio, an anatomical parameter that may provide valuable information for individualized treatment strategies [21].

This study investigated the association between root canal filling quality and the prevalence and volume of apical periodontitis lesions using CBCT in a Chilean population. A secondary objective was to evaluate the relationship between the canal-to-root diameter ratio and the presence and volume of apical periodontitis.

## Materials and methods

### Sample size calculation

The sample size was estimated based on the mean prevalence of 75% for apical periodontitis in root canal-treated teeth as reported in South American CBCT-based studies [22, 23]. The calculation was performed using the formula for cross-sectional studies estimating simple proportions: n = (Z² × p × (1 – p)) / d², where Z is the critical value for a 95% confidence interval (Z = 1.96), p is the expected prevalence (0.75), and d is the acceptable margin of error (0.05). Applying this formula, the minimum required sample size was 288 teeth. To account for potential losses or exclusions during image evaluation, a 20% increase was applied, resulting in a final sample size of 346 teeth. However, the sample size was increased to guarantee more robust and reliable results.

### Study design and sample selection

This retrospective cross-sectional study analyzed CBCT scans from a private radiology clinic in Santiago, Chile. Ethical approval was obtained from the Institutional Research Ethics Committee of UNIGRANRIO University (approval number: 5.615.148). The scans used were requested by other dentists between 2021 and 2024 for reasons not related to this study. Inclusion criteria were: permanent teeth with root canal treatment and coronal restoration, homogeneous root canal obturation without apparent voids, availability of complete CBCT scans with adequate diagnostic quality, free of motion artifacts, distortion, or structural superimposition, and patients aged ≥ 18 years.

Teeth were excluded if they exhibited incomplete root development, marginal bone loss exceeding 4 mm, previous apical surgery, or structural damage such as vertical root fracture, perforation, or external resorption that substantially altered the tooth anatomy. Additionally, teeth with extensive apical periodontitis lesions compromising the cortical bone or involving the maxillary sinus, which prevented accurate volumetric analysis, or the presence of metallic artifacts or prosthetic restorations that impaired adequate visualization were excluded.

### CBCT imaging protocol

Scans were obtained using a Veraviewepocs 3D R100 (J Morita, Kyoto, Japan), operating at 90 kVp, 8 mA, 0.125 mm voxel size, 40 × 40 mm field of view, and 9.3 s exposure. Images were visualized and analyzed using i-Dixel software (J. Morita, Kyoto, Japan).

### Examiner calibration and reliability

Two endodontists (R. A. T. T. and P. A.) were trained and calibrated under the supervision of a senior radiologist with over 15 years of experience (M. T.). Calibration was performed using 100 randomly selected CBCT exams of root canal-treated teeth not included in the study. Interexaminer agreement exceeded 0.70 (Cohen’s kappa), indicating substantial agreement. Disagreements were resolved by consensus or by the radiologist.

### Assessment of the periradicular status and lesion volume

**Fig. 1:**
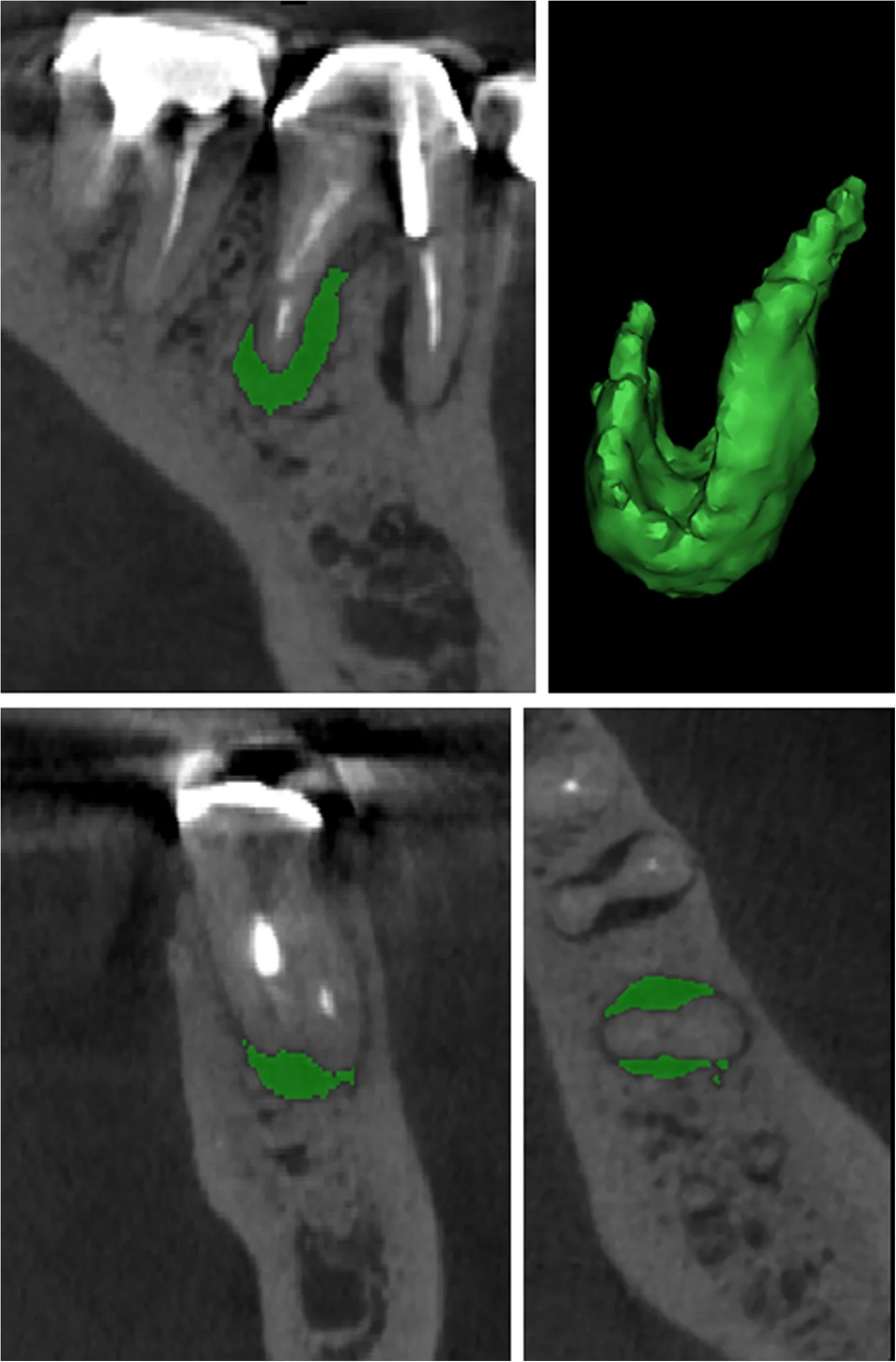
Three-dimensional reconstruction of an apical periodontitis lesion from CBCT scan

Periradicular status was evaluated on axial, coronal, and sagittal CBCT slices. An apical periodontitis lesion was defined as a radiolucency exceeding twice the width of the normal periodontal ligament space with lamina dura disruption [17]. Lesions were segmented using Mimics Innovation Suite 21 (Materialise NV, Leuven, Belgium). The segmentation protocol included: (a) importation of DICOM files and application of density thresholds to isolate low-density areas; (b) manual refinement of masks in axial, coronal, and sagittal planes to exclude anatomical noise; (c) three-dimensional reconstruction of lesion volumes, with results expressed in mm³; (d) exportation of segmented lesions as stereolithography (STL) files for 3D visualization (Fig. 1).

Lesion size was classified according to the CBCT Periapical Volume Index (CBCTPAVI), which categorizes apical periodontitis lesion volumes into seven scores: 0 (0 mm³); 1 (0.01–0.20 mm³); 2 (0.21–0.70 mm³); 3 (0.71–8.00 mm³); 4 (8.01–70.00 mm³); 5 (70.01–100.00 mm³); 6 (> 100.01 mm³) [24].

### Assessment of root canal filling quality

Root canal filling quality was assessed in relation to its extension, considering the radiographic apex on CBCT images. Using high magnification, the distance between the filling terminus and the radiographic apex was measured. Fillings were classified as adequate (terminus at 0–2 mm from the apex) or inadequate (overextended fillings or underfilled canals that were > 2 mm short of the apex). Homogeneity of the filling was not analyzed as an independent variable because only teeth with apparently void-free fillings were admitted based on the inclusion criteria. This methodological decision was crucial to ensure a reliable assessment of the canal-to-root diameter ratio, as the presence of voids could compromise the delineation of the filled canal and root structure, thereby introducing measurement errors.

### Canal-to-root ratio

To evaluate the relationship between root canal enlargement and periradicular status, the ratio between the filled root canal diameter and total root diameter was calculated. Measurements were taken 1 mm short of the apical end of the filling in both the buccolingual and mesiodistal directions. For each root, the largest value (buccolingual or mesiodistal) was selected to represent the canal-to-root diameter ratio, which was then expressed as a percentage.

### Statistical analysis

Data were analyzed using SPSS v.25 (IBM, Armonk, NY, USA). Descriptive statistics included means, standard deviations, and frequencies. Associations between categorical variables and lesion presence were tested using chi-square and odds ratios (OR) with 95% confidence intervals. Logistic regression was performed to evaluate the independent effect of variables such as age, sex, tooth type, and filling quality. Kruskal-Wallis test was used to compare the differences in apical periodontitis lesion volume among tooth types (anterior, premolars and molars). The relationship between canal-to-root diameter ratio and lesion presence was assessed with the Mann–Whitney test, while correlations between canal-to-root diameter ratio and lesion volume were evaluated using Spearman’s correlation coefficient. Statistical significance was set at *p* < 0.05.

## Results

### Sample characteristics

A total of 793 CBCT examinations were initially screened. Of these, 153 were excluded for not fulfilling the eligibility criteria, leaving 640 examinations from 520 patients. These scans had been originally requested for implant planning (*n* = 181), endodontic assessment (*n* = 157), oral surgery (*n* = 130), periodontal evaluation (*n* = 31), and for indications not specified in the referral (*n* = 141). Within these examinations, 830 root canal–treated teeth met the eligibility criteria and constituted the final sample analyzed. This sample comprised 67.3% females and 32.7% males, with a mean age of 50.3 ± 14.7 years (range: 18–91 years). Patients were categorized into three age groups: 18–39 years (27.0%), 40–59 years (47.0%), and ≥ 60 years (26.1%). Teeth were distributed as follows: 50.7% molars, 29.3% premolars, 14.2% incisors, and 5.8% canines (Table 1). In general, 60.8% were maxillary and 39.2% mandibular.

**Table 1:** Distribution of teeth and apical periodontitis lesions by tooth type and arch

Tooth type	Arch	*n* (%)	With lesions *n* (%)	Lesion volume mean ± SD
Incisors	Maxillary	87 (10.5%)	27 (31.0%)	41.18 ± 29.07
	Mandibular	31 (3.7%)	9 (29.0%)	61.07 ± 26.78
Canines	Maxillary	26 (3.1%)	3 (11.5%)	97.58 ± 38.94
	Mandibular	22 (2.7%)	7 (31.8%)	89.61 ± 32.92
Premolars	Maxillary	161 (19.4%)	55 (34.2%)	62.13 ± 70.60
	Mandibular	82 (9.9%)	25 (30.5%)	74.47 ± 37.72
Molars	Maxillary	231 (27.8%)	102 (44.2%)	59.55 ± 47.32
	Mandibular	190 (22.9%)	82 (43.2%)	58.80 ± 42.32
Total	–	830 (100%)	310 (37.3%)	60.51 ± 48.85

### Prevalence of apical periodontitis

Apical periodontitis lesions were observed in 37.3% (*n* = 310) of the root canal-treated teeth. Molars exhibited a significantly higher prevalence of lesions compared with anterior teeth (43.7% vs. 27.7%, *p* < 0.05) and premolars (43.7% vs. 32.9%, *p* < 0.05). No significant difference was observed between anterior teeth and premolars (27.7% vs. 32.9%, *p* = 0.312). Also, no significant differences were found by sex or age (*p* > 0.05).

### Association with root canal filling quality

Adequate fillings were observed in 42.5% of the cases. Lesions were more frequent in inadequately filled teeth (43.8%) than in adequately filled ones (28.6%). Binary logistic regression revealed that adequate fillings were associated with a significantly lower probability of lesion occurrence (OR = 0.514; CI 0.383-0.689; *p* < 0.001), representing a 48.6% reduction in the odds of periradicular disease compared with inadequate fillings (Tables 2 and 3).

**Table 2:** Association between filling quality and lesion prevalence

Filling quality	*n* (%)	Teeth with lesions *n* (%)	Mean lesion volume (mm³)	SD (mm³)	Range (mm³)
Adequate	353 (42.5%)	101 (28.6%)	50.3	39.0	2.7-211.4
Inadequate	477 (57.5%)	209 (43.8%)	65.5*	52.3*	0.3-327.8
Total	830 (100%)	310 (37.3%)	60.5	48.9	0.3-327.8

*p < 0.05 vs. adequate fillings

**Table 3:** Distribution of teeth and apical periodontitis lesions according to filling quality, tooth type, and arch

Tooth type	Arch	Adequate with lesions *n* (%)	Inadequate with lesions *n* (%)	Total *n* (%)	Total with lesions *n* (%)
Incisors	Maxillary	16/61 (26.2%)	11/26 (42.3%)	87 (10.5%)	27 (31.0%)
	Mandibular	7/19 (36.8%)	2/12 (16.7%)	31 (3.7%)	9 (29.0%)
Canines	Maxillary	0/18 (0.0%)	3/8 (375%)	26 (3.1%)	3 (11.5%)
	Mandibular	4/15 (26.7%)	3/7 (42.9%)	22 (2.7%)	7 (31.8%)
Premolars	Maxillary	19/89 (21.3%)	36/72 (50.0%)	161 (19.4%)	55 (34.2%)
	Mandibular	14/50 (28.0%)	11/32 (34.4%)	82 (9.9%)	25 (30.5%)
Molars	Maxillary	27/53 (50.9%)	75/178 (42.1%)	231 (27.8%)	102 (44.2%)
	Mandibular	14/48 (29.2%)	68/142 (47.9%)	190 (22.9%)	82 (43.2%)
Total	–	101/353 (28.6%)	209/477 (43.8%)*	830 (100%)	310 (37.3%)

Data in the two filling-quality columns are given as the number of teeth with lesions / total number of teeth with that filling quality in the same row, followed by the corresponding percentage

### Lesion volume

The mean lesion volume was 65.5 ± 52.3 mm³ in inadequately filled teeth and 50.3 ± 39.0 mm³ in adequately filled teeth; this difference reached statistical significance (*p* < 0.05) (Tables 1 and 2). There was no significant difference in lesion volume among the tooth types (anterior, premolars, and molars). The distribution of periradicular status according to the CBCTPAVI in relation to filling quality is presented in Table 4.

**Table 4:** Periradicular status of root canal–treated teeth as related to the quality of fillings

CBCTPAVI	Adequate (*n* = 128)*	Inadequate (*n* = 177)*	Total (*n* = 305)
1	0 (0%)	2 (0.66%)	2 (0.66%)
2	3 (0.98%)	10 (3.28%)	13 (4.26%)
3	8 (2.62%)	7 (2.30%)	15 (4.92%)
4	23 (7.54%)	12 (3.93%)	35 (11.48%)
5	44 (14.43%)	67 (21.97%)	111 (36.39%)
6	50 (16.39%)	79 (25.90%)	129 (42.30%)

*Percentages are expressed over the total number of lesions (*n* = 305), not within columns

### Canal-to-root diameter ratio

The mean canal-to-root diameter ratio was 0.20 ± 0.07. A weak but significant inverse correlation was found between canal-to-root diameter ratio and lesion volume (Spearman’s ρ = − 0.110, *p* < 0.05), indicating that larger canal diameters were associated with smaller lesion volumes. No significant differences were observed for lesion presence (*p* > 0.05) (Tables 5 and 6).

**Table 5:** Maximum canal-to-root ratio (%) according to tooth type and arch

Tooth type	Maxillary mean ± SD (%)	Range (%)	Mandibular mean ± SD (%)	Range (%)	Total mean ± SD (%)	Total range (%)
Incisors	23.10 ± 7.72	11.67–54.78	17.33 ± 2.55	12.89–22.46	21.59 ± 7.21	11.67–54.78
Canines	19.30 ± 5.17	9.30–31.00	18.86 ± 3.76	12.93–26.48	19.10 ± 4.54	9.30–31.00
Premolars	20.52 ± 8.06	10.44–73.15	19.35 ± 5.34	9.72–45.64	20.13 ± 7.27	9.72–73.15
Molars	19.48 ± 6.07	9.03–50.00	19.72 ± 7.26	9.56–52.01	19.59 ± 6.63	9.03–52.01
Total	–	–	–	–	20.00 ± 6.83	9.03–73.15

**Table 6:** Distribution of canal-to-root ratio (%) by tooth group

Tooth group	11–20%	21–30%	31–40%	41–50%	> 50%
Anterior	94	55	15	1	1
Premolars	159	66	12	6	1
Molars	278	109	27	7	1

## Discussion

This study investigated the association between root canal filling quality and the prevalence and volume of apical periodontitis lesions in Chilean individuals using CBCT. Previous epidemiological studies have largely relied on two-dimensional radiographs or dichotomous CBCT assessments. A strength of this study is the novel application of the CBCTPAVI, a periapical index specifically adapted for CBCT [24], along with the canal-to-root diameter ratio measurement, both employed here for the first time to examine their relationship with the periradicular status. Furthermore, the sample size was the largest reported in CBCT-based studies of this kind, allowing more precise characterization of bone involvement, including small (< 1 mm³) and very large (> 100 mm³) lesions. The results demonstrated that the quality of root canal filling had a significant influence on the periradicular status. Teeth with adequate fillings exhibited a 48.6% lower odds of presenting apical periodontitis lesions (OR = 0.514; 95% CI 0.383–0.689; *p* < 0.001).

The prevalence of apical periodontitis in this study was 37.3%. This figure is within the range documented for some European populations using conventional radiographs: 31.5% in France [25], 39% in Finland [26], and 40% in Belgium [27]. By contrast, the present finding was markedly lower than that reported in South American populations, with 88% in Brazil [22] and 62% in Colombia [23]. Notably, only the Brazilian and Colombian studies used CBCT. Differences among studies may reflect variations in treatment standards, operator experience, and access to advanced techniques. Another factor that may have contributed to the lower prevalence in our study is the exclusion of teeth with voids in the filling mass, a criterion necessary for accurate canal-to-root diameter ratio measurement, but which may have restricted the spectrum of inadequately filled cases included.

The higher prevalence of apical periodontitis lesions observed in molars compared with premolars and anterior teeth aligns with their more complex root canal anatomy, which may hinder effective cleaning and shaping during root canal treatment. This is consistent with findings reported by previous studies [6, 28].

The volumetric analysis provides a more adequate determination of the lesion size than merely the lesion diameter. Most previous studies have relied on dichotomous assessments (presence/absence of AP), whereas the quantification of lesion volume allows for more refined evaluation of disease progression and severity. In the present study, lesions associated with inadequate fillings were significantly larger, suggesting that inadequate treatment not only increases the likelihood of lesion occurrence but also contributes to more extensive periradicular breakdown. This finding is consistent with studies indicating that underfilled canals with unsealed spaces are a key risk factor for posttreatment disease [29, 30]. These observations underscore the role of filling quality as a surrogate marker of overall treatment quality, including canal preparation. Teeth with greater unfilled apical root canal areas are likely to harbor residual bacteria, which may receive nutrients from the periradicular tissue fluids and exudate, grow and sustain apical periodontitis [1].

An inverse correlation was observed between canal-to-root diameter ratio and lesion volume. A higher canal-to-root diameter ratio reflects a larger canal preparation, which permits enhanced irrigation, mechanical debridement, and biofilm disruption, thereby improving disinfection [31–35]. Studies have demonstrated an improved long-term treatment outcome in teeth with large apical preparations [11, 36, 37].

The cross-sectional design and the lack of clinical follow-up limit the ability to establish causality or assess healing outcomes and should be acknowledged as an important limitation of this study. Only teeth with homogeneous, apparently void-free fillings were eligible, a criterion required for reliable canal-to-root diameter measurement. Since teeth with voids are more likely to be both inadequately filled and associated with apical periodontitis, their exclusion may have contributed to the comparatively low prevalence observed and would be expected to attenuate rather than exaggerate the association between filling quality and periradicular disease. The present estimates are therefore likely conservative. Moreover, lesion volume may be influenced by patient-related factors, such as immune response and systemic conditions, which were not accounted for in the analysis. Prospective longitudinal studies encompassing the full spectrum of obturation quality are needed to correlate obturation quality, lesion volume, and clinical outcomes over time.

In conclusion, this CBCT-based study in a Chilean population showed that apical periodontitis affected over one-third of root canal-treated teeth. Adequate root canal fillings were significantly associated with a lower probability of apical periodontitis and smaller lesion volume. An inverse correlation between canal-to-root diameter ratio and lesion volume suggested that greater canal enlargement may favor improved treatment outcome. These findings highlight the importance of high-quality obturation and adequate canal preparation for sustaining long-term periradicular health and provide baseline data for future epidemiological comparisons.

## Data Availability

No datasets were generated during the current study.
